# A systematic review on passive sensing for the prediction of suicidal thoughts and behaviors

**DOI:** 10.1038/s44184-024-00089-4

**Published:** 2024-09-23

**Authors:** Rebekka Büscher, Tanita Winkler, Jacopo Mocellin, Stephanie Homan, Natasha Josifovski, Marketa Ciharova, Ward van Breda, Sam Kwon, Mark E. Larsen, John Torous, Joseph Firth, Lasse B. Sander

**Affiliations:** 1 Medical Psychology and Medical Sociology, Faculty of Medicine, University of Freiburg, Freiburg, Germany; 2 Department of Clinical, Neuro and Developmental Psychology, Vrije Universiteit Amsterdam, Amsterdam, The Netherlands; 3 Experimental Psychopathology and Psychotherapy, Department of Psychology, University of Zurich, Zurich, Switzerland; 4 Department of Psychiatry, Psychotherapy and Psychosomatics, Psychiatric University Hospital Zurich, University of Zurich, Zurich, Switzerland; 5 Black Dog Institute, University of New South Wales, Sydney, NSW Australia; 6 Amsterdam Public Health Research Institute, Faculty of Behavioural and Movement Sciences, Vrije Universiteit Amsterdam, Amsterdam, The Netherlands; 7 Beth Israel Deaconess Medical Center, Harvard Medical School, Boston, MA USA; 8 Centre for Big Data Research in Health, University of New South Wales, Sydney, NSW Australia; 9 Division of Psychology and Mental Health, Manchester Academic Health Science Centre, University of Manchester, Manchester, UK

**Keywords:** Risk factors, Psychiatric disorders

## Abstract

Passive sensing data from smartphones and wearables may help improve the prediction of suicidal thoughts and behaviors (STB). In this systematic review, we explored the feasibility and predictive validity of passive sensing for STB. On June 24, 2024, we systematically searched Medline, Embase, Web of Science, PubMed, and PsycINFO. Studies were eligible if they investigated the association between STB and passive sensing, or the feasibility of passive sensing in this context. From 2107 unique records, we identified eleven prediction studies, ten feasibility studies, and seven protocols. Studies indicated generally lower model performance for passive compared to active data, with three out of four studies finding no incremental value. PROBAST ratings revealed major shortcomings in methodology and reporting. Studies suggested that passive sensing is feasible in high-risk populations. In conclusion, there is limited evidence on the predictive value of passive sensing for STB. We highlight important quality characteristics for future research.

## Introduction

Over 700,000 people worldwide die by suicide every year^1^. In the US, the number of suicide attempts is approximately 25 times higher than the number of deaths by suicide^2^. Suicide is the fourth leading cause of death among young people^1^ and worryingly, rates are rising in this age group, with deaths by suicide increasing by over 45% among adolescents over the last 10 years in the US^3^. The majority of transitions from suicidal ideation to behaviors occur within 12 months from onset^4^.

Research efforts have been extensive in identifying risk factors for suicidal thoughts and behaviors (STB)^5,6^. However, the prediction of STB remains a major challenge^5^. There are several reasons for this. First, the base rate of STB, particularly suicide attempts and deaths, is low, making large sample sizes necessary for developing risk prediction models^5^. Second, research has repeatedly focused on distal risk factors that have little predictive value for the timing of a suicide attempt^5^. Third, the remarkable fluctuations in suicidal ideation, as observed in several studies based on ecological momentary assessment (EMA), exacerbate the problems around the prediction of STB^7,8^. While initial suicidal thoughts often occur years before an attempt, the final steps towards attempts, including the final decision, are taken within days to minutes^9^. Fourth, STB result from a complex interaction of various risk factors that is insufficiently understood^10–12^. These challenges are mirrored by the finding that clinicians’ risk assessments cannot categorize their patients’ risk states in clinically meaningful ways^13^. In order to navigate these complexities, it has been suggested that research should focus on short-term risk prediction for STB instead of distal factors^5^.

New and promising approaches have emerged for studying and potentially identifying shifts in risk states for STB in real-time, such as EMA using smartphone-based surveys and passive sensing^14^. EMA requires active participation^15^, which can potentially be burdensome for participants, especially during longer assessment periods^8^. In contrast, passive sensing passively collects digital markers (e.g., geolocation, sleep parameter) via smartphones or wearable devices^14,16,17^. A range of sensors can be used to capture a variety of data points, depending on the characteristics of a device. Predominantly, physiological (e.g., electrodermal activity), social (e.g., call logs, apps used), and behavioral (e.g., sleep duration) signals can be captured via sensors^18^. Compared to questionnaires only capturing a certain moment in time, passive sensing allows an objective and continuous flow of data^14,17^.

Several viewpoint articles have delineated promises and challenges associated with the application of passive sensing within the field of suicide prevention^14,19–21^. Nonetheless, a systematic review of the current state of research remains absent. Against this backdrop, we aimed to systematically synthesize the current evidence on passive sensing for the prediction of STB. This review encompassed the exploration of three core facets: (1) the potential of passive sensing data to enhance STB prediction, (2) the discriminative efficacy of various sensor types, and (3) an examination of the statistical analysis methodologies documented in the literature. Additionally, we synthesized findings pertaining to the feasibility of employing passive sensing for the assessment of STB.

## Methods

This report follows the Preferred Reporting Items for Systematic Reviews and Meta-Analyses guidelines^22^ (see Supplementary Table 1). All procedures were pre-registered (https://osf.io/hzxua) and reported in a study protocol^23^. Due to the infancy of the field, we decided to broaden the scope of the review by a) additionally including feasibility papers and study protocols, and by b) additionally including prediction papers with non-clinical samples, regardless of the presence of STB.

### Eligibility criteria

We included studies with participants with and without STB at baseline, without restrictions on age, gender, or current treatment status. Studies had to address the use of passive data through smartphones or wearables in the context of STB. We included all passive sensor modalities. We included studies regardless of whether participants received treatment. Studies were eligible if they reported a quantitative measure of STB as an outcome; we excluded studies restricted to non-suicidal self-injurious behavior. Studies were eligible if they reported results either on the association between passive data and STB or on the feasibility of passive sensing. Study protocols were additionally included to provide a perspective on the research currently being processed. We included peer-reviewed articles without any restrictions on language or publication date.

### Search strategy

The following databases were searched from inception to June 24, 2024: MEDLINE, PubMed, Embase, PsycINFO, and Web of Science. The search string is displayed in the study protocol^23^. We validated the search string against seven hand-searched relevant articles; the search string reached a coverage rate of 100%. The relevant articles were screened using the online tool Covidence. In the first step, two independent researchers screened all titles and abstracts against the eligibility criteria. After obtaining all relevant full texts, a second screening of the articles was conducted. Conflicts were resolved in discussion with a third reviewer (LS) where necessary.

### Data extraction

All relevant information was extracted from the final set of studies and double-checked by an independent researcher, including the following variables: Study identification items, study design, sociodemographic and clinical sample characteristics, data collection device, type and collection frequency of passive data, sensors used, assessment of STB, assessment length, and analysis methods. Any findings about an association between the passive data and STB or the predictive value of the passive data for STB were extracted. Descriptive results on feasibility of passive sensing were extracted as secondary outcomes.

### Risk of bias assessment

Two independent researchers assessed risk of bias using the Prediction model Risk Of Bias ASsessment Tool (PROBAST) tool^24^. The PROBAST tool is specifically designed to assess potential sources of bias in studies for the development or evaluation of prediction models. It covers the following domains: participants, predictors, outcome, and analysis. Conflicts were resolved in discussion with a third researcher.

### Data analysis

The results were described narratively. In the field of prediction modeling, there are a variety of performance measures, which complicates the reporting and interpretation of the findings. Therefore, we selected and reported performance measures of prediction models in line with a decision tree developed by WVB and MC^25^ that provides a hierarchy of the most meaningful performance measures, along with a guideline for the interpretation of the predictive value (i.e., very good, good, acceptable, or poor). These were reported in the section “Main Findings of Prediction Studies”. Where deemed helpful for the understanding of the findings, additional measures were reported. A meta-analytic synthesis of predictive validity was not possible due to the heterogeneity in the identified studies.

## Results

### Search results

**Fig. 1:**
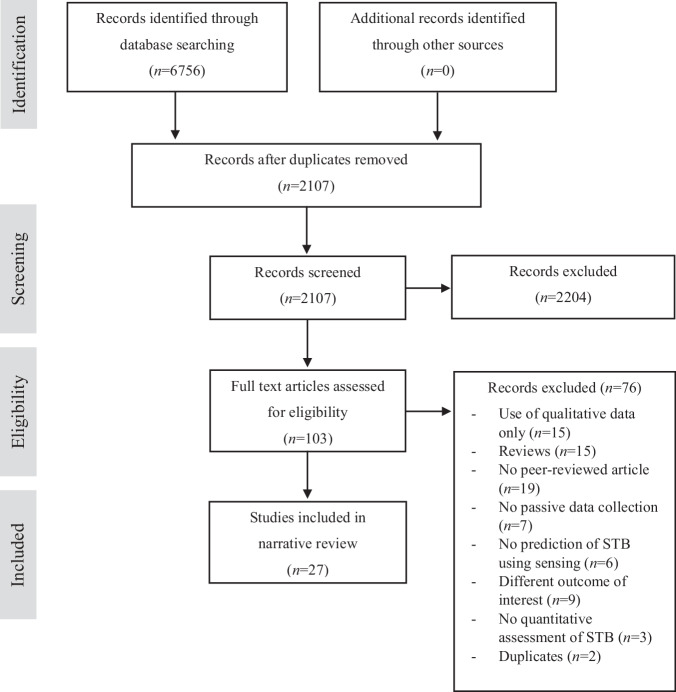
Study selection flowchart. STB suicidal thoughts and behaviors.

The search yielded 2107 unique records (see Fig. 1 for the study selection process). In a first step, two independent reviewers screened the titles and abstracts against eligibility criteria. In the next step, they screened the 103 potentially relevant full texts and resolved any conflicts with an independent researcher, revealing 27 eligible articles. This included eleven studies investigating the predictive value of passive sensing for the prediction of STB^26–36^, ten trials focusing on the feasibility of passive sensing^28,37–45^, and seven study protocols^46–52^. One article reported on two studies: a feasibility investigation and a prediction study^28^. All papers were published between 2019 and 2024.

### Characteristics of prediction studies

The study characteristics are displayed in Table 1. Sample sizes ranged from 25^32^ to 2881 participants^30^. More than half of the studies were based on clinical populations, i.e., participants were in inpatient or outpatient treatment^26,29,32–36^. All non-clinical studies had larger sample sizes than clinical studies, at least 281^27^. One study recruited adolescents^33^, whereas the other studies were in adult populations.

**Table 1:** Characteristics of prediction studies

Author (year)	Predictors (device name)	Sensors/device analytics (study length)	Assessment of STB (frequency)	Main statistical analysis	Sample characteristics
Barrigon et al.^34^	Distance traveled, time spent at home, steps, app usage (Smartphone app: eB2)	GPS, device usage, activity (measured by Google Fit), accelerometer (6 months)	Electronic health records: suicide attempts or psychiatric emergency department visits (NA)	Unsupervised machine learning model (Heterogeneous Mixture Model)	225 adult outpatients with a history of suicide attempt or ideation
Bertrand et al.^26^	Sleep characteristics (Wrist device: GENEActiv)	Accelerometer (14 days)	C-SSRS quick screen (2), QIDS-SR-16 item 12 (2), MADRS item 10 (2)	Spearman’s rank order correlations, multiple linear regression	76 adult outpatients with bipolar disorder
Coyne et al.^27^	Smartphone app use, including social media (Smartphone apps: Moment or Cronicle)	Screentime (14 days)	SBQ-R (1)	Multilevel models	281 adults from the general population
Czyz et al.^35^	Sleep, heart rate, steps (Wrist device: Fitbit)	NA (56 days)	Two EMA items: duration and intensity of ideation (4 per day)	Mixed-effects classification and regression trees	102 young adult emergency department patients with acute suicidality
Dogrucu et al.^28^	Retrospective: GPS, browser history, call logs, text messages, contacts, social media (Smartphone app: Moodable)	GPS, microphone, calls, quantitative features of contacts, text messages, social media (14 days)	PHQ-9 item 9 (1)	Machine learning algorithms (KNN, SVM, RF)	335 adults (general population), recruited via online platform (Mechanical Turk)
Haines-Delmont et al.^29^	Sleep, steps, smartphone usage including social media (Smartphone apps: SwiM, Apple Health kit; wrist device: Fitbit)	Accelerometer, Wi-Fi, screentime (7 days)	C-SSRS (3)	Machine learning algorithms (KNN, RF, SVM), logistic regression	66 adult inpatients (acute mental health)
Horwitz, Czyz et al.^30^	Sleep, physical activity, heart rate (Smartphone; wrist device: Fitbit)	Accelerometer, gyroscope, light sensor (92 days)	PHQ-9 item 9 (2)	Three-step hierarchical logistic regression with suicidal subsample (*n* = 217)	2881 first-year medical residents
Horwitz, Kentopp et al.^31^	Sleep, physical activity, heart rate (Smartphone; wrist device: Fitbit)	Accelerometer, gyroscope, light sensor (92 days)	PHQ-9 item 9 (2)	Machine learning algorithms (RF, ENR)	2459 first-year medical residents
Kleiman et al.^32^	Physiological distress (electrodermal activity)^b^ (Smartphone app: LifeData; wrist device: Empatica Embrace 2)	Accelerometer, gyroscope, thermostat, electrodermal activity sensor (Inpatient stay + 28 days after discharge)	Three EMA items (6 per day)	Multilevel models	25 adult inpatients with acute suicidality
Salvatore et al.^36^	Circadian activity (Wrist device: Motionlogger)	Accelerometer (3 days)	EMA item: wish to die (3; one per day)	Linear regression	83 adult outpatients with a current major depressive episode and either a bipolar or major depressive disorder
Sheridan et al.^33^	Heart rate variability (wrist device)	Light sensor (7 days)	C-SSRS (3)	Multilevel models	51 adolescent inpatients with acute suicidality

*C-SSRS* Columbia-Suicide Severity Rating Scale, *EMA* ecological momentary assessment*, GPS* Global Positioning System, *MADRS* Montgomery Åsberg Depression Rating Scale, *PHQ-9* Patient Health Questionnaire, *QIDS-SR* Quick Inventory of Depressive Symptomatology*, SBQ-R* Revised Suicidal Behavior Questionnaire, *STB* suicidal thoughts and behaviors, *Wi-Fi* wireless fidelity.

Ten studies investigated behavioral signals^26–32,34–36^, including sleep, physical activity, smartphone usage, and activity patterns such as time spent at home. Five examined physiological signals using wrist devices^30–33,35^, including the heart rate (variability) and the electrodermal activity. Three explored social signals^27–29^, including social media use, text messages, call logs, and phone contacts. Overall, smartphone usage (7/11)^27–32,34^, sleep characteristics (6/11 studies)^26,29–31,35,36^, and physical activity (5/11 studies)^29–31,34,35^ were studied most frequently. Studies that asssessed physiological or social signals typically combined them with behavioral signals; only one study investigated a physiological signal (heart rate variability) alone^33^. To collect passive data, three studies used smartphones only^27,28,34^, four used wrist devices only^26,33,35,36^, and four studies used both^30–32^. Four studies used research grade wearables^26,32,33,36^, and four used commercial wearables^29–31,35^. Only one study reported the sampling frequency of the device; it was a frequency of 4hz in electrodermal activity assessment^32^. Several studies reported pre-processing regarding the time resolution of the data; one study reported a summary for the past 14 days^28^, seven studies reported a daily-level, and two an hourly-level aggregation^32,33^. Two studies provided a detailed listing of the device sensors and analytics^32,33^. The passive data collection interval varied between 7 days^29,33^ and 6 months^34^.

Studies differed in how they assessed STB. Several studies assessed suicidal ideation using single items of validated scales, (e.g., item 9 from the PHQ-9)^26,28,30,31^. Furthermore, the Columbia-Suicide Severity Rating Scale (C-SSRS) was frequently used^26,29,33^, and one study used the Suicide Behaviors Questionnaire (SBQ)^27^. The studies using the C-SSRS and the SBQ used a composite score, which did not differentiate between suicidal thoughts and behaviors. One study assessed suicide attempts and emergency department visits via electronic health records^34^. Studies divided the outcome into the presence or absence of STB (8/11 studies)^28–35^ and/or evaluated STB on a severity level (5/11 studies)^26,27,32,33,36^. Eight studies assessed STB between one and three times in total, and two studies used EMA items to collect momentary data on suicidal ideation several times a day^32,35^.

Regarding statistical analysis strategies, two studies calculated correlations and/or regression analyses^26,36^, four studies applied multilevel models^27,30,32,33^, and five studies used machine learning algorithms^28,29,31,34,35^.

### Main findings of prediction studies

Seven studies developed predictive models based on multiple predictors^28–32,34,35^; details on the study designs are displayed in Table 1. In an EMA study with suicidal inpatients (*n* = 25), Kleiman and colleagues^32^ investigated the electrodermal activity (i.e., autonomic arousal) until 28 days after discharge. While physiological data did predict suicidal ideation both concurrently (according to a performance score on the percentage of investigated performance indices that speak for best fit: 0.00–1.14%; conditional R^2^ = 0.76–0.92; moderate to strong relationship) and prospectively (performance score: 0.00–40.80%; conditional R^2^ = 0.78–0.94; moderate to strong relationship), multilevel prediction models performed poorest when only physiological data was included (i.e., poorer than models with only self-reported affective states, with performance score: 29.99–99.95%; conditional R^2^ = 0.80–0.96; strong relationship). When physiological data was added to self-reported affect, the fit improved for the outcome severity of suicidal ideation in seven out of eight models (performance score: 59.40–100.0%; conditional R^2^ = 0.80–0.83; strong relationship vs. only self-report performance score: 54.33–86.05%; conditional R^2^ = 0.80–0.81; strong relationship) and worsened for the presence of suicidal ideation in seven out of eight models (performance score: 27.90–83.02%; conditional R^2^ = 0.92–0.96; strong relationship vs. only self-report performance score: 29.99–99.95%; conditional R^2^ = 0.92–0.96; strong relationship). In an EMA study with suicidal emergency department patients (*n* = 102), Czyz and collegues^35^ assessed sleep duration, steps, and resting heart rate. While the passive sensing data predicted next-day suicidal ideation, the model performance was poor (mean AUC = 0.56). When combined with EMA data, the model performance was good (mean AUC = 0.84); however, passive sensing did not improve the prediction compared to EMA only (mean AUC = 0.84; good performance).

No other studies assessed momentary STB. Haines-Delmont and colleagues assessed actively collected (i.e., sociodemographic variables, mood assessments, journaling) and passively collected data (sleep, steps, smartphone usage) in a sample of adults admitted to acute mental health wards^29^. The optimal prediction model for STB was the k-nearest-neighbors algorithm; the performance was poor (AUC = 0.65). In a prospective study, Barrigon and collegues^34^ passively monitored adult outpatients (n = 225) with a history of suicidal ideation or attempt for six months via their smartphones. Individual daily activity profiles were created based on distance traveled, steps, time at home, and app usage, to predict risk events (suicide attempts or emergency department visits requiring psychiatric assessment) within a 1-week window. The machine learning model based on these profiles predicted short-term risk with a good model performance (AUC = 0.78). The study by Horwitz and colleagues^30,31^ assessed sleep, physical activity, heart rate, and mood using a Fitbit and a mobile app in first-year medical residents during the first three months of internship. In participants from an earlier wave (academic years 2018–2020, *n* = 2881), the three-step hierarchical logistic regression indicated that the passive data did not have incremental predictive validity for the prediction of suicidal ideation over baseline demographic and clinical variables (step one AUC 0.735, acceptable performance; vs. step two 0.739, acceptable performance; BIC step one 1125.0 vs. step two 1155.2)^30^. In a later cohort (academic year 2020–2021, *n* = 2459), a machine learning model^31^ that only used daily self-reported mood performed better (i.e., a sensitivity of 0.70 and a specificity of 0.73, AUC = 0.74; acceptable performance) than the model that additionally incorporated passive data (i.e., sensitivity of 0.67 and a specificity of 0.69, AUC = 0.70; acceptable performance). Dogrucu and colleagues^28^ assessed active data (i.e., a brief voice sample) combined with passive social media account data and smartphone usage data (i.e., GPS, browser history, call logs) that was collected retrospectively from smartphones in a non-clinical adult sample (*n* = 335). The machine learning algorithm predicted presence vs. absence of suicidal ideation with a good performance of F1 = 0.85 using active and passive data; no alternative models were reported.

Four studies^26,27,33,36^ investigated associations of single passive data modalities with STB. In a sample of adult outpatients diagnosed with bipolar disorder (*n* = 76), Bertrand and colleagues^26^ assessed sleep characteristics via wrist actigraphy. Using three different measurements of STB and no correction for multiple testing, they found associations of several sleep variables with one of the STB measures each, inconsistent across instruments and timepoints. Sheridan and colleagues^33^ investigated the heart rate variability using a wrist device in acutely suicidal adolescents (*n* = 51). They found an increase in the high-frequency component (i.e., higher parasympathetic activity) in patients that had at least a 25% decrease in STB within the 7-day study period. Salvatore and collegues^36^ assessed the circadian activity using a wrist device in adult outpatients with a current major depressive episode and either a major depression or bipolar disorder. They found no association between the self-rated wish to die and circadian activity. This included the amplitude (β = −0.19, *p* > 0.05), daytime activity (β = −0.19, *p* > 0.05), mesor (β = −0.19, *p* > 0.05), and nighttime activity (β = −0.11, *p* > 0.05). However, they found a positive correlation between these activity indices and the self-rated wish to live and the wish to die. Coyne and colleagues^27^ assessed smartphone app usage in a non-clinical cohort of adolescents (*n* = 281). They found that in girls, a higher use of entertainment apps was associated with an increased risk for STB (composite score; β = 0.23–0.25, *p* < 0.001); in boys, the use of reading apps (e.g., Reddit) was associated with an increased higher risk for STB (β = 0.28–0.29, *p* < 0.001).

### Risk of bias in prediction studies

Risk of bias in prediction model studies was assessed using the PROBAST tool. The ratings per domain are displayed in Table 2 (see Supplementary Table 2 for detailed ratings). Most studies (7/11) had a high risk of bias in the domain of participants, mostly because the eligibility criteria and reasons for exclusion were unclear. Across all studies, the bias potential was low in the domain of predictors. Furthermore, the bias potential regarding the outcome was high in most trials (9/11). STB were often assessed using single items, drastically limiting the reliability and validity of the assessment^53^. An additional potential for bias was introduced by choosing arbitrary cut-offs to determine high or low suicide risk, or insufficient information on how this was calculated. One study assessed change by 25% in C-SSRS scores^33^, which does not account for measurement error like in the Reliable Change Index^54^. Risk of bias was mostly high in the domain of analysis (8/11 studies). Most studies were either not pre-registered or had vague pre-registration, failing to provide clear information on how predictors were assessed in a time-resolved manner, selected, and analyzed. Studies rarely reported on how they dealt with missing data. There was often imbalance in the sample regarding the proportion of participants with the outcome; frequently, it was unclear how imbalance was managed. Another critical issue in the analysis domain was the insufficient validation of prediction models. Only three studies used a train-test split approach or cross validation of the model^29,31,35^. This failure to test on out-of-sample observations exposes to the risk of over-fitting and does not allow conclusions to be drawn on the generalizability of the prediction results.

**Table 2:** Risk of bias of prediction studies

Study	Participants	Predictors	Outcome	Analysis
Barrigon et al.^34^	+	+	+	-
Bertrand et al.^26^	+	+	-	+
Coyne et al.^27^	-	+	-	+
Czyz et al.^35^	+	+	-	+
Dogrucu et al.^28^	-	+	-	-
Haines-Delmont et al.^29^	-	+	-	-
Horwitz, Czyz et al.^30^	-	+	-	-
Horwitz, Kentopp et al.^31^	-	+	-	-
Kleiman et al.^32^	+	+	+	-
Salvatore et al.^36^	-	+	-	-
Sheridan et al.^33^	-	+	-	-

*PROBAST* Prediction model Risk OF Bias ASsessment Tool, *ROB* risk of bias. + indicates low ROB; - indicates high ROB.

### Characteristics of feasibility studies

In total, ten studies investigated the feasibility of passive sensing in the context of STB. A summary of all relevant study characteristics is displayed in Table 3. Nine studies examined behavioral signals^28,37–42,44,45^, six studied social signals^28,37,38,41,44,45^, and three investigated physiological signals^39,40,43^. Data collection lasted between one retrospective session^37,38^ and a mean of 229.4 days (SD = 168), varying across participants^39^. Sample size varied from 50 subjects^43^ to 1709 subjects^45^, with five studies investigating fewer than 100 subjects^37,39,41–43^. Samples consisted of adults in eight studies^28,37–41,44,45^ and adolescents in two studies^42,43^. Six studies examined clinical samples^39–44^, three investigated general samples^28,37,38^, and one study included students and a sample of patients with and without STB^45^. The definition of feasibility varied across trials; studies focused on user experience, adherence to passive sensing technology (i.e., wearing the device or keeping a sensing app on a smartphone), and the willingness to share data.

**Table 3:** Characteristics of feasibility studies

Author(s) (year)	Feasibility definition (measure)	Device (name)	Sensors/device analytics (study length)	Sample characteristics	Findings
Bruen et al.^41^	User experience (qualitative interview), rate of Facebook sharing	Smartphone app (SwiM), wrist device (Fitbit)	Accelerometer, Wi-Fi, screentime (7 days)	80 adult inpatients	The majority provided positive feedback on Fitbit. A total of 19% of participants provided access to Facebook data.
Dogrucu et al.^28^	Willingness to disclose passive smartphone data, social media (survey)	Not applicable: no passive data collection	Not applicable, no passive data collection	202 adults, recruited via online platform (Mechanical Turk)	Participants reported more willingness to share active (e.g., voice recordings) compared to passive data (e.g., browser history).
Glenn et al.^42^	Participation rate, device adherence, user experience and acceptability (interview), burdensomeness and impact on care (clinician survey)	Wrist device (Actiwatch), smartphone for EMA	Accelerometer (28 days)	53 adolescents after discharge from acute psychiatric care for suicide risk	Participation rate of 25%. Participants wore the wrist device on 76% of days. 71% of participants stated that wearing the wrist device was comfortable. Clinicians found their study participation was minimally burdensome.
Jiang et al.^40^	Device adherence (time worn) and barriers to adherence (questionnaire), acceptability (questionnaire)	Wrist device (Fitbit), smartphone for EMA	No information (56 days)	106 young adults with last-month suicide attempt or last-week ideation	102/106 enrolled participants wore the Fitbit at least 1 minute, with a mean of 53.6% of minutes and on 65.9% of days within the monitoring period. Satisfaction mean 3.89 (SD 1.19), 92.6% found it comfortable, 18.1% reported interference with daily activities, 85.1% would wear it outside a study. Most frequent barriers: water-related activity (45.7%), forgot to wear it (39.4%).
Kleiman et al.^43^	Device adherence (hours worn, correct use of event marker), user experience (Wearable Computer Comfort Rating Scale; qualitative interview)	Wrist device (Empatica E4), smartphone for EMA	Accelerometer, thermostat, electrodermal activity sensor, light sensor (duration of hospitalization, mean days = 10.7, SD = 13.86)	50 adolescent inpatients with acute STB	Wrist device worn for M = 20.3 hours per day, and at 95% of all days; 0.46% of event marker presses were accidental. Most items on discomfort rated below 5/10. Qualitative findings: complaints about the monitor (e.g., too clunky), neutral, and positive views. 7 participants stopped wearing the monitor before discharge.
Ortiz et al.^39^	Device adherence (days worn), predictors of adherence	Smart ring (Oura Ring)	Accelerometer, gyroscope, infrared optical pulse measurement (varying study length; mean 229.4 days, SD = 168)	87 adults with bipolar disorder	Participants wore the ring on 79.5% of days. Being in the “perfect adherence” group was associated with female gender and a history of suicide attempt (attempt: 11/21 (52.4%) vs. no attempt: 16/66 (24.2%,), *p* < 0.05)
Porras-Segovia et al.^45^	Recruitment rates, retention rates	Smartphone app (eB²), EMA	Accelerometer, gyroscope, GPS, Bluetooth, Wi-Fi, calls, app usage, screentime (60 days)	139 patients with history of STB; 1224 outpatients without STB; 346 psychology students	Recruitment rates were 87% in patients with history of STB and 85% in patients without STB. Rates of installed eB² app were 74% and 72% in patients with and without history of STB. Retention rates of eB² were >65% in all groups.
Porras-Segovia et al.^44^	Participation and retention rates, dropout from app, satisfaction survey	Smartphone app (eB²), EMA	Accelerometer, gyroscope, GPS, Bluetooth, Wi-Fi, calls, app usage, screentime (180 days)	393 adult outpatients	Participation rate of 80%; the retention rate for eB^2^ was 87.8% after 1 month and 46.6% after six months. Mean satisfaction with eB^2^: 7/10. Most valued aspect was the control of physical activity (14%); 23% reported technical issues.
Tlachac et al.^37^	Willingness to share optional passive data vs. active data	Smartphone app: (EMU); for sensing and active data	Text messages, calendar, call log, GPS, usernames for Twitter, Instagram, Google Maps (one session, retrospective data)	70 participants recruited via Mechanical Turk	Participants were unlikely to share passive (compared with active data): 31 (44.3%) text messages, 28 (40.0%) calendar, 25 (35.7%) call logs, 14 (20.0%) GPS, 14 (20.0%) Twitter name, 0 Instagram.
Tlachac et al.^38^	Willingness to share optional passive data for depression and suicidal ideation screening	Smartphone app: (EMU); for sensing and active data + similar web app	Text logs, call logs, contacts, calendar entries, GPS history (one session, retrospective data)	302 tertiary students (33 opted for the smartphone app and only they were asked for passive data)	Of 33 students opting for the smartphone app, 21 (63.6%) shared GPS, 11 (33.3%) calendar/contacts, and 10 (30.3%) call/text logs. Of 171 students who reached the Twitter page, 47 (27.5%) had an account, which 16/47 (34.0%) were willing to share.

*EDA* electrodermal activity, *GPS* Global Positioning System, *SI* suicidal ideation.

### Main findings of feasibility studies

Several studies investigated the user experience or satisfaction with passive sensing technology. Adolescent inpatients with acute suicidality rated discomfort in wearing a wrist device (Empatica E4), scoring below 5/10 in most items of the Wearable Computer Comfort Rating Scale^43^. Qualitative findings revealed complaints about the device (e.g., too clunky), neutral responses, and positive views (e.g., liking to help research)^43^. In a sample of adolescents who wore a wrist device (Actiwatch) after discharge from acute psychiatric care for suicide risk, 71% found wearing the device comfortable; clinicians also reported that the study participation (that additionally involved EMA) had a neutral to positive impact on the patients^42^. In a study by Jiang and collegues^40^ with young adult suicidal patients, 92.6% found the Fitbit comfortable, 18.1% reported that it interfered with daily activities, and 85.1% stated that they would wear it outside the research context. The satisfaction with wearing it was rated 3.89 (SD = 1.19) on a 0–5 scale. The most frequent barriers were water-related activities (45.7%), forgetting to wear it (39.4%), and other activities (20.2%). Another study found that the majority of adult inpatients provided positive qualitative feedback on the use of a Fitbit^41^. A study that investigated a passive sensing smartphone application in adult outpatients found a mean satisfaction with the app of 7/10^44^. In addition, the most frequently reported positive aspect of the app was that it helped control physical activity; the most frequent negative aspect was technical problems^44^.

Regarding adherence to passive sensing technology, two studies on adolescents with STB found that they wore a wrist device on more than 75% of days^42,43^. A total of 7 out of 50 participants stopped wearing the device before discharge^43^. The study by Jiang and collegues with young adult suicidal patients recruited via emergency departments^40^ found that in an 8-week monitoring phase, a wrist device was worn on 65.9% of days, and in 53.6% of minutes. Adherence decreased during the monitoring period, with 37.3% of participants stopping to wear the device before the end of the study. In a study with adult bipolar patients^39^, a smart ring was worn on 79.5% of days; the likelihood for perfect adherence was higher in patients with a history of suicide attempt. A study with an adult outpatient sample^44^ and a study with adult patients and university students^45^ investigated how many participants kept a passive sensing smartphone app installed and did not withdraw from the study. They found retention rates of >65% across all groups after two months^45^, 74% after three months, and 47% after 6 months^44^.

In a sample with tertiary students (*n* = 302)^38^, only 33 (10.9%) opted to share data for depression and suicidal ideation screening via a smartphone app as opposed to a web app and were asked to share optional passive data via the app. Of these, 21 (63.6%) shared GPS, 11 (33.3%) calendar/contacts, and 10 (30.3%) call/text logs. Of 171 students who reached the Twitter page, 47 (27.5%) had an account, which 16/47 (34.0%) were willing to share. In a sample recruited online via Mechanical Turk (*n* = 70)^37^, participants were more willing to share active compared to passive smartphone data. Active data included questionnaires for depression and anxiety screening, demographic data, scripted and unscripted audio, and sharing rates ranged from 78.6% to 100% for these modalities. In contrast, a total of 31 (44.3%) shared text messages, 28 (40.0%) calendar, 25 (35.7%) call logs, 14 (20.0%) GPS, 14 (20.0%) Twitter name, 0 (0%) Instagram. In an online survey study by Dogrucu and colleagues^28^, where the sample was intended to be representative of the general population, participants reported a higher willingness to share active data (e.g., a brief voice sample) compared with passive data (e.g., browser history). A study with adult inpatients found that beyond compulsory passive data collection using Fitbit, 80% of participants did not provide optional access to their Facebook data^41^; the majority of reported reasons for opting out were not having an account (56%) or currently not using it (21%).

### Upcoming studies

In total, seven study protocols planning to investigate the predictive validity of passive sensing for STB were identified (see Supplementary Table 3 for a detailed overview)^46–52^. All planned studies have a longitudinal prospective design. They will all investigate behavioral signals, while two will also include physiological signals^48,49^ and three will examine social signals^47,48,51^. Most studies^47–52^ plan to investigate sleep characteristics; four studies will additionally assess other variables such as physical activity^47–49,51^. Sels and colleagues provided detailed information about the sensors used and the collection frequency of data for each sensor in their protocol^51^. Brown and colleagues^49^ reported that data will be available at a one-minute resolution (i.e., physical activity, sleep, heart rate) and Victor and colleagues^52^ reported a sampling frequency of approximately 30hz (i.e., accelerometers to derive sleep data). The other protocols did not provide detailed information about the frequency and method of aggregating the data. The majority of study protocols designs reported an EMA assessment of STB^47–52^. Data collection is planned between 14 days^50^ and 60 months^47^. One study reported non-clinical recruitment at schools^46^; all other studies plan to recruit participants from clinical settings. Four studies will only include patients with suicidal ideation or attempts^47,49,51,52^.

## Discussion

This is the first systematic review to address the predictive validity and feasibility of passive sensing for the prediction of STB. Our results indicate that the current evidence base does not permit conclusions about the value of passive sensing modalities for predicting STB. Prediction studies in this nascent field of research are highly heterogeneous in the selection of predictors, samples, modeling strategies, and outcomes. Furthermore, due to the explorative nature of the research so far, there are multiple sources of potential bias and shortcomings in the reporting of methods and results. Thus, the predictive value of passive sensing data remains inconclusive. Nevertheless, preliminary results on models incorporating self-reported outcomes with passive sensing data are broadly in line with machine learning approaches using other data modalities^55^. Early feasibility studies suggest that passive sensing might be feasible in clinical populations, in terms of user experience and adherence in terms of wearing devices or keeping mobile apps. Moving forward, we propose several avenues for future research to further explore the potential of passive sensing in the prediction of STB.

The prediction studies identified in this review indicated that passive sensing may have no incremental value over active data in predicting STB. Out of four studies investigating the incremental predictive value of passive data over self-reported data, only one study—a study that performed short-term prediction of suicidal ideation based on EMA – found an incremental value of passive sensing (i.e., electrodermal activity) in the prediction of suicidal ideation severity in suicidal inpatients^32^. However, physiological data worsened the model fit for the occurrence of suicidal ideation^32^. Another study predicting the short-term presence or absence of suicidal ideation in suicidal patients did not find an incremental value of passive data over EMA^35^; two other studies did not identify an incremental value of physiological data over daily mood assessments in the prediction of STB at three-month follow-up in medical interns^30,31^. Algorithms based only on passive modalities did predict STB^32,34,35^, usually with a lower model performance compared with active data only^32,35^. Notably, in one study, events including suicide attempts and emergency department visits were predicted in the short-term with a good model performance using activity profiles from passive smartphone sensing^34^. Other studies found correlations of post-test STB with specific app usage in adolescents^27^, sleep characteristics^26^, and the high-frequency component in heart rate variability^33^. There was a high risk of bias overall, with severe shortcomings in the study design and transparency in reporting.

Nevertheless, preliminary feasibility studies suggest that passive sensing for STB is feasible in high-risk populations. Patients with STB and/or mental disorders reported moderate to high levels of comfort while wearing wrist devices^40,42,43^ and using a sensing app^44^, with sufficient adherence to these devices, including a smart ring^39,40,42,43^. However, participants from the general population seem to be more willing to share active data compared to passive data^28,37,41^. Potentially, individuals might be reluctant to share passive data under some circumstances such as a non-clinical setting or the possibility to opt out of specific passive data.

The findings of this review hold important implications for future research. First and foremost, studies should address general methodological limitations by adhering to the Transparent Reporting of a multivariable prediction model for Individual Prognosis Or Diagnosis (TRIPOD) checklist^56^ and by avoiding potential sources of bias according to the PROBAST tool^24^. Studies should rigorously report the following aspects both in pre-registrations and research reports: eligibility criteria and reasons for exclusion from analyses, missing data handling, details regarding the selection of predictors and feature extraction algorithms, pre-processing steps of passive sensing data, a detailed list of sensors, the sampling rate and method for each sensor, as well as the model performance. Concerning the sampling strategy, studies should select participants with the aim of achieving a balanced sample containing a substantial proportion of participants or time points with and without the outcome of STB, which is necessary for model development and comparison to a control/naive predictor^24^. Authors should report how they dealt with potential class imbalance in classification tasks. In addition, studies should avoid choosing arbitrary cut-offs for differentiating participants into high- and low-risk^24^. Finally, it is crucial to evaluate models using external data^24^.

Second, it is crucial to prioritize the valid and reliable assessment of STB. Single-item measures, as frequently used in the reviewed studies, may not have sufficient psychometric properties^53^. In addition, suicidal ideation and suicidal behaviors should be differentiated (e.g., the subscales of the CSSR-S) instead of using a composite score including both of them, as they are likely to have different risk trajectories^57^. Furthermore, most studies have assessed STB at single time points. Given the high fluctuations in STB^8^, it might be fruitful to additionally investigate the short-term prediction by assessing these outcomes using EMA^5^.

Third, research on passive sensing in the context of STB should consider different application settings and target groups. Target groups identified in this review included acutely suicidal patients^32,33^, patients with a history of STB^34^, groups at risk of developing STB (i.e., people in mental health treatment^26,29,36^ or medical interns^30,31^), as well as the general population^27,28^. Each application area presents unique challenges. The short-term prediction of STB within individuals with acute STB seems promising both clinically and methodologically. Clinically, intensive monitoring is desirable in this vulnerable target group, while methodologically, a high frequency of time points with STB is necessary for developing predictive algorithms. Developing algorithms for predicting suicide attempts might however prove challenging because of the low rates of occurrence of this outcome and the low signal-to-noise ratio of passive sensing data. Transfer-learning on models initially developed for a different but closely related source domain^58^ might be one of the possible workarounds to this methodological hurdle. Another application area of passive sensing could be universal screening for STB; again, studies should address sample imbalance in samples from the general population. Another aspect to consider in the application setting is the use of passive sensing variables as the sole predictors versus combining them with EMA data for predicting STB. Passive data alone might be especially useful for longer monitoring periods due to the low participant effort, but it remains unclear whether passive data alone has sufficient predictive value. Combining EMA and passive sensing holds promise for the longitudinal prediction of STB, making use of the advantages of both assessment strategies, including additional data such as nonresponse^59^ or response latency to questionnaire items^60^. Regarding the choice of devices for assessing predictors, it is important to consider their real-world applicability in different settings. Participants from the general population seem more willing to share actively assessed data compared to passive smartphone usage data^28,37,41^; however, this might not generalize to indicated or selective prevention, such as risk monitoring in individuals with acute suicidal ideation or a recent suicide attempt. Furthermore, the applicability of passive monitoring may be influenced by the availability of devices (i.e., higher availability of smartphones compared with wearables), requiring a balance between data quality and scalability. It is crucial to further consider the applicability of passive monitoring for STB beyond research settings, particularly with regards to how an individual’s awareness of STB monitoring via passive devices could influence behaviors in ways that would affect the reliability of risk detection in real-world settings.

Several limitations of this review should be considered. First, in line with the protocol, the search string aimed to identify prediction studies and might have missed some relevant feasibility studies. Second, the heterogeneity of reviewed papers did not allow a meta-analytic pooling of results. Third, the current results on social signals are limited to quantitative features, such as number of calls or social media application usage. However, contents of social media posts might be highly relevant for the prediction of STB, as indicated in language processing models^61–63^.

In conclusion, the field of passive sensing of STB is currently in its infancy, with the first studies published in recent years. The selection of predictors has been largely explorative, and existing studies – whilst pioneering in their own right - have major methodological shortcomings consistent with first-in-field research. The identified study protocols indicate that studies conducted in the following years may be designed with further rigor to hopefully reveal whether passive data is suitable to predict STB. Future studies should rigorously follow reporting guidelines and pre-register their procedures. Alongside this, the clinical implications of applying such approaches in real-world settings should be considered, to develop tools with maximal utility for helping to detect and ultimately prevent STB among vulnerable populations.

## Supplementary information

Supplementary Information

## Data Availability

The extracted data will be available via OSF with publication (https://osf.io/psk85/files/).
